# Chronic modulation of AMP-Kinase, Akt and mTOR pathways by ionizing radiation in human lung cancer xenografts

**DOI:** 10.1186/1748-717X-7-71

**Published:** 2012-05-18

**Authors:** Yaryna Storozhuk, Toran Sanli, Sarah N Hopmans, Carrie Schultz, Tom Farrell, Jean-Claude Cutz, Gregory R Steinberg, James Wright, Gurmit Singh, Theodoros Tsakiridis

**Affiliations:** 1 Translational Radiation Biology Laboratory, McMaster University, Hamilton, Ontario, Canada; 2 Juravinski Cancer Center, McMaster University, Hamilton, Ontario, Canada; 3 Department of Oncology, McMaster University, 699 Concession Street, Hamilton, Ontario, Canada, L8V 5C2; 4 Department of Pathology and Molecular Medicine, McMaster University, Hamilton, Ontario, Canada; 5 Department of Medicine, McMaster University, Hamilton, Ontario, Canada; 6 Department of Medical Physics and Applied Radiation Science, McMaster University, Hamilton, Ontario, Canada

**Keywords:** Lung cancer, ATM, p53, 4-EBP1, p21^cip1^

## Abstract

****Introduction**:**

Earlier, we showed that in cancer cells, AMP-activated kinase (AMPK) participates in a signal transduction pathway involving ATM-AMPK-p53/p21^cip1^ which is activated by ionizing radiation (IR) to mediate G2-M arrest and enhanced cytotoxicity. We also observed that AMPK modulates ATM expression and activity and the IR response of the Akt-mTOR pathway. Since the ATM, AMPK and Akt pathways are key targets of novel radio-sensitizing therapeutics, we examined the chronic modultion of expression and activity of those pathways by IR alone in xenograft models of lung cancer.

****Methods**:**

Immuno-compromised mice were grafted with human lung A549 and H1299 cells, were treated with a single fraction of 0 or 10 Gy, and left to grow for 8 weeks. Extracted tumors were subjected to lysis and immunoblotting or fixation and immunohistochemical analysis.

****Results**:**

IR inhibited significantly xenograft growth and was associated with increased expression of Ataxia Telengiectasia Mutated (ATM) and enhanced phosphorylation of two ATM targets, H2Ax and checkpoint kinase Chk2. Irradiated tumours showed increased total AMPK levels and phosphorylation of AMPK and its substrate Acetyl-CoA Carboxylase (ACC). IR led to enhanced expression and phosphorylation of p53 and cyclin dependent kinase inhibitors p21^cip1^ and p27^kip1^. However, irradiated tumours had reduced phosphorylation of Akt, mTOR and it‘s target translation initiation inhibitor 4EBP1. Irradiated xenografts showed reduced microvessel density, reduced expression of CD31 but increased expression of hypoxia-induced factor 1A (HIF1a) compared to controls.

****Conclusion**:**

IR inhibits epithelial cancer tumour growth and results in sustained expression and activation of ATM-Chk2, and AMPK-p53/p21^cip1^/p27^kip1^ but partial inhibition of the Akt-mTOR signaling pathways. Future studies should examine causality between those events and explore whether further modulation of the AMPK and Akt-mTOR pathways by novel therapeutics can sensitize lung tumours to radiation.

## **Introduction**

In tumor cells ionizing radiation (IR) activates within minutes the protein kinase B (Akt) and mammalian Target of Rapamycin (mTOR) pathway leading to radio-resistance and tumor survival [1]. Akt and mTOR are established effectors of tyrosine kinase receptors such as EGF receptor (EGFR), which modulates the activity of these molecules through a pathway involving phosphatidylinositol 3-kinase (PI3k) and phosphoinositide-dependent kinase 1 (PDK1) [2]. Akt kinase acts as a main activator of mTOR, up regulation of which is known to occur by at least two different steps: i) phosphorylation and inhibition of Tuberous Sclerosis Complex 2 (TSC2), that inactivates GTPase activity of the GTP-binding protein Rheb leading to mTOR activation [3] and ii) stimulation of mTOR activity through phosphorylation of PRAS40, a member of mTORC1, one of the two functional mTOR complexes, which also includes mLST8/Gbl and the scaffold protein Raptor [4]. To date, extensive published work demonstrated the impact of mTOR on cell growth, cancer cell proliferation and resistance to cytotoxic agents [5] mTORC1 regulates multiple growth and gene expression pathways and specifically stimulates mRNA translation through phosphorylation and activation of the ribosomal p70S6-kinase (p70^s6k^) and phosphorylation-induced inhibition of the translation initiation inhibitor eIF4E binding protein 1 (4EBP1) [5].

Recently, we showed that IR activates acutely the energy sensor and tumor suppressor AMP-activated kinase (AMPK) pathway, an evolutionally-preserved kinase that mediates a metabolic checkpoint on cell cycle when cells are under stress [6]. AMPK is an effector of Liver Kinase B 1 (LKB1), a tumour suppressor mutated in Peutz-Jeghers syndrome, which is associated with benign and malignant epithelial tumors [7]. AMPK is a heterotrimeric enzyme of α, β and γ subunits that senses low energy levels through AMP binding on the γ subunit and is regulated by phosphorylation of the α subunit on Thr172 [8]. AMPK inhibits anabolic processes and protein synthesis by inhibition of mTORC1 through different mechanisms including, i) Ser1387 phosphorylation and activation of TSC2, leading to enhanced Rheb GTPase activity and mTOR inhibition and ii) by Raptor phosphorylation [9]. In addition, AMPK mediates cell cycle checkpoints through induction of p53 and the cyclin-dependent kinase inhibitors (CDKI) p21^cip1^ and p27^kip1^ leading to cell cycle arrest [6,10].

We have suggested that, apart from its metabolic action, AMPK is activated by IR and may be a mediator of DNA damage signals. We implicated AMPK in the mediation of IR-induced signal transduction through an Ataxia Telengiectasia mutated (ATM)-AMPK-p53-p21^cip1^ pathway to facilitate G2/M cell cycle arrest and mediate radiosensitization [6]. However, the effects of IR on AMPK subunit expression and chronic regulation of its activity have not been examined in human tumours. Furthermore, the levels of expression and activation of the Akt and mTOR pathways have not been analyzed extensively in irradiated tumours long after treatment. Here, we analyzed in two different human non-small cell lung cancer xenograft models the effects of a single fraction of IR on the long term expression and activation of the AMPK and the Akt-mTOR pathways, as well as their upstream regulator ATM.

## **Methods and materials**

### **Animal treatments**

Balb/c immune-compromised nude mice were obtained from Charles River (Mississauga, Ontario, Canada). At five weeks of age, animals were injected into the right flank with 1x10^6^ A549 or H1299 human lung adenocarcinoma cells. Once tumours reached 100 mm^3^, animals were equally divided into non-irradiated (control: 0 Gy) or ionizing radiation (IR: 10 Gy) treated groups (n = 6 per group). Tumour volume was measured every 3 days with calliper according to the formula: V = Lenght*Width*Height*0.5236. Eight weeks after treatment, tumours were extracted and snap-frozen in liquid nitrogen for lysis, total protein extraction and immunoblotting or were formalin fixed and paraffin embedded for immunohistochemistry (IHC) analysis. Tumour lysates were prepared from frozen tumours that were sectioned, mechanically homogenized in RIPA (Radio-Immunoprecipitation Assay) buffer and manually processed with Dounce homogenizer for total protein extraction.

### **Animal irradiation**

After appropriate dosimetry, conformal IR treatment (10 Gy) was delivered to xenografts with a clinical radiotherapy unit while animals were anaesthetized and housed in a Plexiglas tube equipped with High-Efficiency-Particulate-Air-(HEPA) filters.

### **Immunoblotting**

Immunoblotting was performed as described previously [6]. Antibodies for total AMPKα, P-AMPKα (Thr172), P-ACC(Ser79), ATM, γH2AX (Ser139), P-Chk2 (Thr68), P-p53 (Ser15), p27^kip^, p21^waf/cip^, mTOR, P-mTOR(Ser2448), Akt, P-Akt (S473), P-Akt (Thr308), P-4EBP1(Thr37/46), CD31 and HIF1α were purchased from Cell Signalling Technology (Mississauga, Ontario, Canada). Antibodies against p53 and β-actin were supplied by Millipore (Etobicoke, Ontario, Canada).

### **Immunohistochemistry**

Four μm thick tumour sections were mounted onto slides, deparaffinised, followed by antigen retrieval, blocking with goat serum and incubated with primary antibody against P-AMPKα (Thr172) (1:200), anti-CD31 (1/500) dilution overnight and processed as described earlier [11].

### **Statistical analysis**

Quantitation and normalization of immunoblotting results was pursued for all xenograft lysates and antibodies (12 per tumour type and 6 per condition, Control vs irradiated). All density values of each immublotting band were first normalized against a value that for each blot was defined by the average density of the 6 control (untreated) lysates in each tumor type. Mean and SE values were determined after this normalization.

Paired t-test was performed to analyze the results from immunoblotting experiments using SPSS software (SPSS, Chicago, IL). Results are presented as Mean ± SEM. Statistical significance was determined at p < 0.05 (*).

## **Results**

### **Effects of IR on lung cancer xenograft growth**

**Figure 1:**
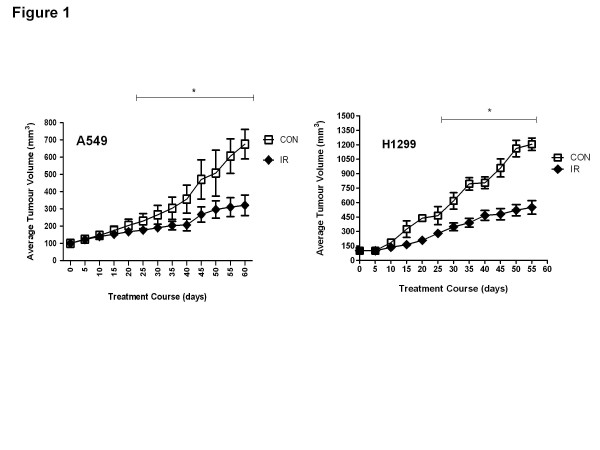
**Ionizing radiation (IR) suppresses A549 and H1299 lung cancer tumour growth*in vivo*.** Twelve four week old male balb/c nude male mice were grafted with A549 or H1299 cells and left to 100 mm³ and were treated with a single fraction of 0 Gy (Control) or 10 Gy R. Tumour volume was monitored every 3 days for a period of 8 weeks. Representative graph of average tumour volume are shown. *p < 0.05 compared with control tumour volume.

Within 15 days after IR treatment, xenografts began to show differences in growth kinetics that became statistically significant by day 25 (Figure 1). At the end of the 8 week period irradiated tumours were on average 67 ± 3.4 % (A549) and 70 ± 4.2 % (H1299) smaller than their control (non-irradiated) counterparts.

### **Effects of IR on the ATM expression and activity**

**Figure 2:**
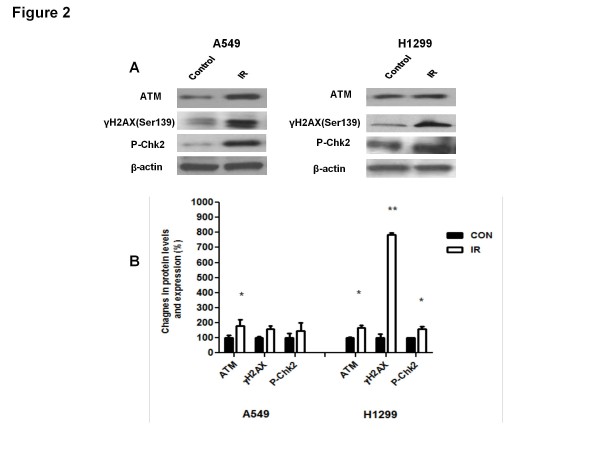
**Ionizing radiation (IR) induces sustained stimulation of the DNA damage response pathway**. ( **A**) Tumour tissue extracted from Control and IR–treated animals were subjected to immunoblotting analysis using ATM, P-Chk2 and γH2AX (Ser139) antibodies. Anti-actin was used as a loading control. Representative immunoblots from 6 independent experiments are shown. ( **B**) Immunoblot densitometric values are shown as percent change in protein expression relative to the control group (*p < 0.05).

We examined the effects of IR on the total protein levels and the activity of ATM. Eight weeks after IR treatment A549 xenografts exhibited significantly increased levels of total ATM protein (Figure 2a). To evaluate the activity of ATM we assessed the phosphorylation levels of two established targets of this kinase, histone H2AX and the checkpoint kinase Chk2. In both A549 and H1299 xenografts we detected increased levels of phosphorylated H2AX (γH2AX) in the irradiated tumours compared to untreated control tumours that were significantly higher in H1299 xenografts (Figure 2A-B). Similarly, irradiated A549 and H1299 xenografts showed increased Chk2 phosphorylation (P-Chk2). That was statistically significant in H1299 but not in A549 xenografts when all tumours were analyzed (Figure 2).

### **Chronic regulation of expression and activity of AMPK by IR**

**Figure 3:**
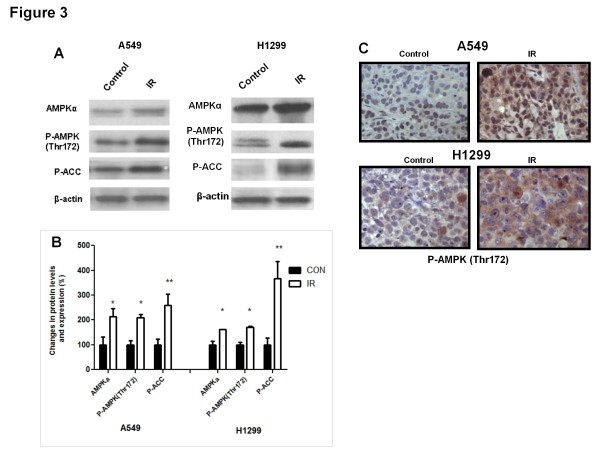
**Ionizing Radiation (IR) upregulates AMPK expression and activity in A549 and H1299 lung xenografts*in-vivo.*** ( **A**) Control and IR-treated tumours were subjected to immunoblotting analysis using AMPKα, P-AMPK (Thr172), and P-ACC antibodies. Anti-actin was used as a loading control. A representative immunoblot of 6 independent experiments is shown. ( **B**) Immunoblot densitometric values are shown as percent change in protein expression relative to the control group p (*p < 0.05; **p < 0.001). ( **C**) A549 and H1299 tumours were fixed and immunohistochemistry analysis was performed using a specific P-AMPK antibody.

In recent studies with tissue cultures of A549 cells, we observed that within 24-48 h IR stimulates expression of AMPK subunits at both the mRNA and protein level [25]. For that we examined here whether those effects of IR could be sustained in xenografts long after IR delivery. The levels of total AMPKα, P-AMPK and P-Acetyl CoA Carboxylase (P-ACC), a substrate of AMPK indicating AMPK kinase activity, were examined in control and irradiated A549 and H1299 tumours. Basal levels of total AMPK α subunit increased in irradiated xenografts along with activation of the enzyme marked by phosphorylation on Thr172 residue (Figure 3A-C). P-ACC levels were also significantly higher in tumours collected from irradiated xenografts compared to control (Figure 3 A and B). Figure 3B shows the quantitation results of immunoblotting experiments of 6 xenografts per group. To examine whether increased levels of P-AMPK (Thr172) signals are indeed attributed to cancer cells, rather than to the surrounding tumor microenvironment, we have performed immunohistochemistry analysis of xenografts using anti-P-AMPK (Thr172) antibody (Figure 3C). In those experiments we also observed significant increases in P-AMPK in irradiated tumour cells compared to controls that distributed both cytoplasm and nuclei of tumor cells of A549 origin but mainly in cytoplasm of H1299 tumour cells.

### **Regulation of steady state levels of p53 and CDKIs by IR**

**Figure 4:**
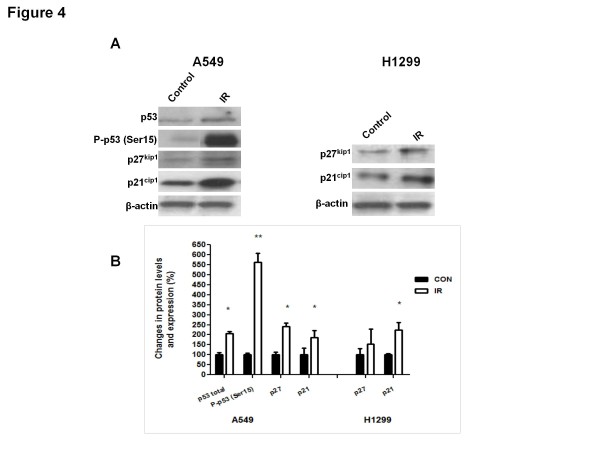
**Ionizing radiation (IR) activates cell-cycle regulatory proteins in lung cancer tumour xenographts.** ( **A**) Tumour tissue extracted from Control and IR–treated animals were subjected to immunoblotting analysis using p53, P-p53 (Ser15), p27^kip^, and p21^waf/cip^ antibodies. Anti-actin immunoblotting was used as a loading control. A representative immunoblot from 6 independent experiments is shown. (**B**) Immunoblot densitometric values are shown as percent change in protein expression relative to the control group (*p < 0.05; **p < 0.001).

To examine the effects of IR treatment on cell cycle checkpoint regulators, lysates of control and IR-treated xenografts were probed with anti-p53, P-p53 (Ser15), p27^kip1^ and p21^cip1^ antibodies. Figure 4A-C shows that a single fraction of IR induces a sustained significant increase, of p27^kip1^ and p21^cip1^ levels in irradiated A549 and H1299 tumours. We analyzed total and phosphorylated (P-) p53 levels specifically in A549 tumours only as H1299 tumours lack p53 expression. Interestingly, we detected highly significant increase in total and phosphorylated (Ser15: 5.5-fold increase) p53 levels in irradiated tumours.

### **IR mediates a long term suppression of the Akt-mTOR pathway**

**Figure 5:**
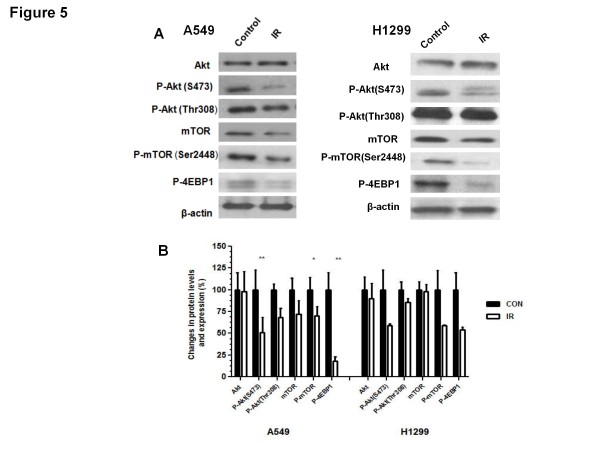
**Ionizing Radiation (IR) inhibits Akt-mTOR pro-survival pathway in A549 and H1299 lung carcinoma xenografts**. ( **A**) Lysates from Control and IR treated tumours were subjected to immunoblot analysis using Akt, P-Akt (S473), P-Akt (Thr308), mTOR, P-mTOR (Ser2448) and P-4EBP1 antibodies. A representative immunoblot from 6 independent experiments is shown. ( **B**) The immunoblot densitometric values are shown as percent change in protein expression relative to the control group (*p < 0.05; **p < 0.001).

We did not detect significant differences in the total Akt levels between control and irradiated tumours (Figure 5). However, we observed that IR caused a sustained reduction in the levels of P-AktS473 in both A549 and H1299 xenografts that reached significance in A549 but not in H1299 tumours. A trend for reduced P-AktT308 levels was also detected in irradiated tumours of both types but that was not statistically significant in either of them (30.0 + 6.4% and 55.0  + 10.9% vs 15.0 ± 4.3% and 42.0 ± 2.3% decrease for T308 and S473 phosphorylation in A549 and H1299, respectively) (Figure 5B, D). Consistently, both IR-treated tumour types showed reduced P-mTOR (Ser2448) levels without a significant change in total-mTOR levels. Irradiated xenografts of the two lung cancer types showed reduced levels of phosphorylation of 4EBP1 (P-4EBP1) indicating reduced mTOR activity (reduction by 81.0 ± 4.75% and 47.0 ± 3.20% in A549 and H1299 xenografts, respectively) (Figure 5A-B).

### **Levels of microvasculature and hypoxia markers in irradiated xenografts**

**Figure 6:**
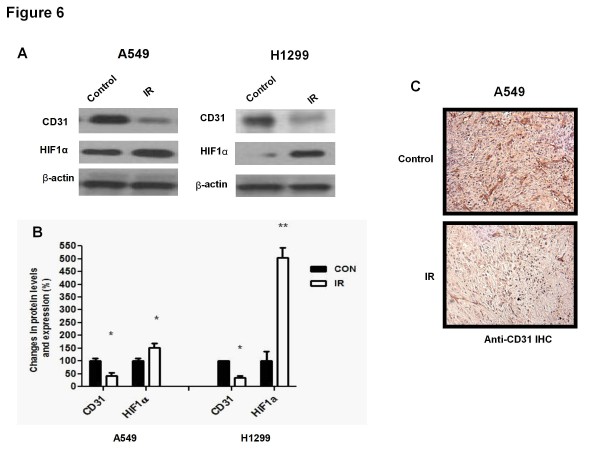
**Ionizing Radiation (IR) downregulates CD31 and enhances HIF1α levels in human lung cancer tumours.** ( **A**) Lysates from control and irradiated tumours were subjected to immunoblot analysis using CD31 and HIF1α antibodies. A representative immunoblot from 6 independent experiments is shown. ( **B**) Average normalized densitometric values are shown as percent change in protein expression relative to the control group (*p < 0.05; **p < 0.001). ( **C**) A549 tumours were fixed and immunohistochemistry analysis was performed using a specific anti-CD31 antibody.

Since hypoxia is known to modulate tumour IR responses and ATM activity, we examined the levels of the endothelial protein CD31, as a marker of microvasculature density, and those of HIF1α, as marker of hypoxia, in control and irradiated xenografts from both lung cancer A549 and H1299 xenografts. Figure 6A and B illustrates representative immunoblots and quantitation of results from all xenografts. Both types of irradiated xenografts showed significantly reduced levels CD31 and increased levels of HIF1α in comparison to untreated tumours (Figure 6A, B). We performed immunohistochemistry experiments with the antibody against CD31 to verify whether indeed the reduced expression of CD31 levels corresponded to a reduced density of microvessels in irradiated tumours. All six tumours per group were analyzed. Figure 6C shows representative images from these experiments illustrating a significantly reduced density of microvessels in the irradiated A549 tumours.

## **Discussion**

The Akt-mTOR pathway is an established mediator of radio-resistance and novel biological inhibitors of the two kinases are shown to sensitize tumour cells to IR [12,13]. On the other hand, AMPK is an emerging metabolic and genomic stress sensor that is also a promising target of novel cancer therapeutics such as the anti-diabetic agent metformin. Metformin inhibits cancer cell proliferation and we have shown that it has radio-sensitizing properties in lung cancer in*-vitro*[6] These notions suggest a need to understand in depth the effects of IR on the expression and activity of the Akt-mTOR and AMPK signaling pathways in tumours in order to understand better tumour radiation biology and assist in a rational development of new effective radio-sensitizers. Here we analyzed the effects of a single fraction of therapeutic IR (10 Gy) on the steady state levels of expression and activity of AMPK and Akt pathway members. Tumours were extracted and analyzed 8 weeks after radiation as this is a typical protocol in pre-clinical radio-sensitizer studies. Two different NSCLC tumour models with distinct molecular defects (A549: K-Ras (G12S) oncogenic mutant and truncated LKB1-null but wild-type p53 vs H1299: p53-null, wild-type K-Ras and LKB1) were used to examine whether detected chronic response of the AMPK-p53/CDKIs and Akt-mTOR pathways to IR apply in lung cancer types with diverse oncogenic genotypes.

Treatment of human lung xenografts with a single fraction of IR (10 Gy) caused an expected significant inhibition of tumour growth kinetics (Figure 1). Since our earlier studies suggested that AMPK is an effector of ATM [6] and other work pointed to direct modulation of Akt activity by ATM [14] we explored the effect of IR on ATM expression and activity. Interestingly, we observed increased total ATM levels and increased phosphorylation of two ATM targets, histone H2AX and Chk2 (Figure 2). Both events are well described acute effects of IR. Enhanced levels of H2AX have also been described in human tumours 24 h after a clinical dose of radiotherapy of 2 Gy [15]. However, our results suggest a sustained increased activity of ATM-γH2AX DNA damage response pathways long after exposure to IR treatment which can be responsible for the increased activity of the AMPK pathway discussed below.

The detection of a sustained enhancement of AMPKα protein levels and activity in tumours long after IR is a novel finding in this study (Figure 3). Irradiated tumours had significantly higher levels of total and phosphorylated AMPK as well as P-ACC suggesting maintained enhanced expression and activity of the enzyme. Since we and others have shown that AMPK is a transducer of ATM signals [6,16] sustained activation of AMPK would be an expected finding in the presence of ATM activation. However, our results also showed increased AMPKα protein levels, suggesting that IR drives AMPKα gene expression. In recent studies with lung (A549) and breast cancer cells (MCF7 and MB-231), we observed that within 24 and 48 hour IR enhances not only the activity of AMPK but also the levels of mRNA and protein of AMPKα, β and γ subunits [17] indicating that IR regulates AMPK gene expression at both the transcriptional and the translational level. Those results suggested that IR stimulates significantly AMPK gene expression within 24 – 48 h that is maintained long after the genotoxic insult is delivered. The specific mechanism and transcription factors involved in these events remain to be elucidated but studies suggest involvement of the p53-dependent stress-responsive genes Sestrin 1 and 2 [18]. The regulation of AMPK gene expression and activity in response to IR is likely a universal phenomenon in epithelial tumour cells. Similar to observations in lung cancer xenografts, we have observed sustained enhancement of total and phosphorylated AMPK α subunit levels in xenografts of PC3 prostate cancer cells also, a cell line that lacks expression p53 (see Additional file 1: Figure S 1). Therefore, overall our results suggest that IR triggers acute and chronic expression of AMPK genes as well as activation of this enzyme that is likely universal in epithelial cancer cells and is independent of p53. Currently, we analyze the exact role of sestrin genes in these processes.

Importantly, we observed that irradiated tumours maintain significantly increased levels of total and phosphorylated p53 and of CDK inhibitors p21^cip1^ and p27^kip1^ (Figure 4). We also detected in irradiated tumours highly increased level of p53-Ser15 phosphorylation a post-translational modification believed to contribute to a greater stability of this protein [14]. These results support the notion that IR activates the p53/CDKI signaling pathways in tumours in a sustained fashion probably through increased expression, phosphorylation and stabilization of p53 and increased levels of CDKIs p27^kip1^ and p21^cip1^ (Figure 4). The p53-p21^cip1^ pathway is an established target for ATM [19] and AMPK [6,8] both of which were suggested to phosphorylate p53. Earlier, we showed that induction of p53 and p21^cip1^ in response to IR is dependent on AMPK and that AMPK activity is required for the mediation of IR-induced G2-M checkpoint and IR cytotoxicity [6]. AMPK may indeed mediate the inhibitory effects of IR on xenograft growth through regulation of p53 and CDKIs. Similar to our earlier observation on the acute response of p21^cip1^ to IR in A549 and H1299 cell cultures [6], the induction of this CDKI in irradiated xenografts does not appear to depend on p53 as it was observed in p53-null H1299 xenografts also (Figure 4 A).

IR is known to mediate a rapid activation of Akt [20] and recent studies showed that ATM can function as an activating Akt kinase that phosphorylates rapidly Akt-S473 [21]. Despite that, and the detection of increased ATM activity in radiated xenografts (Figure 2), we observed significantly reduced levels of Akt-S473 phosphorylation in both types of lung cancer xenografts and a trend for reduced AktT308 phosphorylation. Consistently, mTOR phosphorylation was partially reduced and so was the activity of this key enzyme indicated by lower 4EBP1 phosphorylation that was more significant in A549 tumours (Figure 5). We have obtained similar results in PC3 prostate cancer xenografts (see Additional file 1: Figure S 1) indicating that these are likely universal responses of human epithelial tumours to IR that are independent of K-Ras mutation status and LKB1 or p53 function. One could contribute the suppressed mTOR activity in xenografts on the enhanced AMPK activity. However, the mechanism of reduced phosphorylation of Akt remains unclear and needs to be elucidated by future studies. Nevertheless, the concept of Akt inhibition in tumours by agents that activate the AMPK pathway has been described in earlier studies by our group and others [22,23]. It is possible that in irradiated tumours conditions develop, long after delivery of IR, that attenuate signal transduction between ATM and Akt leading to suppression of Akt and mTOR activity despite enhanced ATM activation. In irradiated tumours the combined effects of sustained increased expression of AMPK-p53-p21^cip1^/p27^kip1^ pathway, that is shown to lead to inhibition of cell cycling, and inhibition of Akt-mTOR-4EBP1 pathway, known to lead to gene transcription and translation, may be capable of mediating an effective anti-proliferative action in those tumours, which may be adequate to mediate the cytotoxic action of IR [13]. Future studies should examine causality in the relationship between these events.

Our observation of sustained ATM activity in irradiated tumours is a significant finding of the present study. Since ATM is suggested to be a common regulator of the activity of the AMPK-p53/p21^cip1^/p27^kip1^ and Akt-mTOR-4EBP1 pathways [6,14], future work should address the mechanism of this sustained activation of ATM in irradiated tissues. It is possible that ATM activation is the result of sustained, IR-induced DNA damage or genomic instability that remains in tumours long after irradiation. Other mechanisms of ATM activation have been described, including hypoxia. Since IR is known to damage tumour vascular supply one could hypothesize that the sustained ATM activity of irradiated tumours may be the result of hypoxia developing in these tissues rather than sustained DNA damage. Conceivably, the reduced vascular supply and CD31 expression we observed in irradiated xenografts here would be responsible for local tumour hypoxia and the enhanced expression of HIF1α we observed (Figure 6). Interestingly, Cam et al. [24] showed that in hypoxic conditions ATM mediates phosphorylation of HIF1α leading to activation of this molecule and inhibition of mTORC1.

## **Conclusions**

This study explored in tumours the long-term regulation by IR of two key tumour suppression or growth pathways that are targets of promising therapeutics. Despite established acute activation of both the AMPK and Akt-mTOR pathways by IR, irradiated tumours showed a sustained expression and activation of the AMPK-p53/p21^cip1^/p27^kip1^ but inhibition of the activity of the Akt-mTOR-4EBP1 pathway. This was associated with increased expression and sustained activity of the upstream regulator of the two pathways ATM that may be associated with the development of hypoxia in irradiated tumours or with potential genomic instability. These molecular responses of irradiated tumours do not appear to be dependent on typical oncogenic molecular defects detected in lung cancer involving K-Ras, LKB1 or p53 status. The findings of this study provide a basis to understand better the chronic regulation of these key pathways by IR alone. IR causes a favorable but partial modification of the activity of the studied pathways. Additional modulation of those pathways with targeted therapies may be able to improve further radiotherapy responses in lung cancer.

## **Competing interests**

The authors declare no competing interests.

## **Authors’ contributions**

YS pursued some of the animal handling and treatments, carried out the majority of the analysis of the xenograft tissue and helped draft the manuscript. TS aided in the animal handling and treatment studies and helped draft the manuscript. SNH pursued most of the animal grafting, care and radiation treatment handling. CS helped deliver animal radiation. TF performed the radiation dosimetry study and supervised animal radiation. J-CC helped optimize the immunohistochemistry protocols and reviewed slides. GS, JW and GRS provided scientific support. TT conceived the study, directed the study design, supervised all experimental work and prepared the manuscript. All authors have read and approved the final manuscript.

## Supplementary Material

Additional file 1: Figure S1 * The effects of ionizing radiation on PC3 prostate cancer xenografts.* PC3 cells were grafted into the flanks of balb/c nude mice and were treated with or without a single fraction of 10Gy IR. The average tumour volumes from each group were measured and expressed as the mean±SE of 6 animals per group. **A.** Extracted PC3 tumours were lysed and subjected to western blotting with antibodies against the AMPK pathway. **B.** Normalized densitometry values of the results from A. (mean ±SE) of 6 animals per groups are shown. **C.** Extracted PC3 tumours were lysed and subjected to immunoblotting with antibodies against the Akt-mTOR pathway. **D.** Normalized densitometry values of the results from C. (mean ±SE) of 6 animals per groups are shown. Click here for file
