# Supplementary material for: Chronic modulation of AMP-Kinase, Akt and mTOR pathways by ionizing radiation in human lung cancer xenografts
Source: Radiat Oncol. 2012 May 18;7:71. doi: 10.1186/1748-717X-7-71 (PMC3439705; doi:10.1186/1748-717X-7-71)
Supplement: Additional file 1: Figure S1 — The effects of ionizing radiation on PC3 prostate cancer xenografts. PC3 cells were grafted into the flanks of balb/c nude mice and were treated with or without a single fraction of 10Gy IR. The average tumour volumes from each group were measured and expressed as the mean±SE of 6 animals per group. A. Extracted PC3 tumours were lysed and subjected to western blotting with antibodies against the AMPK pathway. B. Normalized densitometry values of the results from A. (mean ±SE) of 6 animals per groups are shown. C. Extracted PC3 tumours were lysed and subjected to immunoblotting with antibodies against the Akt-mTOR pathway. D. Normalized densitometry values of the results from C. (mean ±SE) of 6 animals per groups are shown. [file 1748-717X-7-71-S1.ppt]

## Slide 1
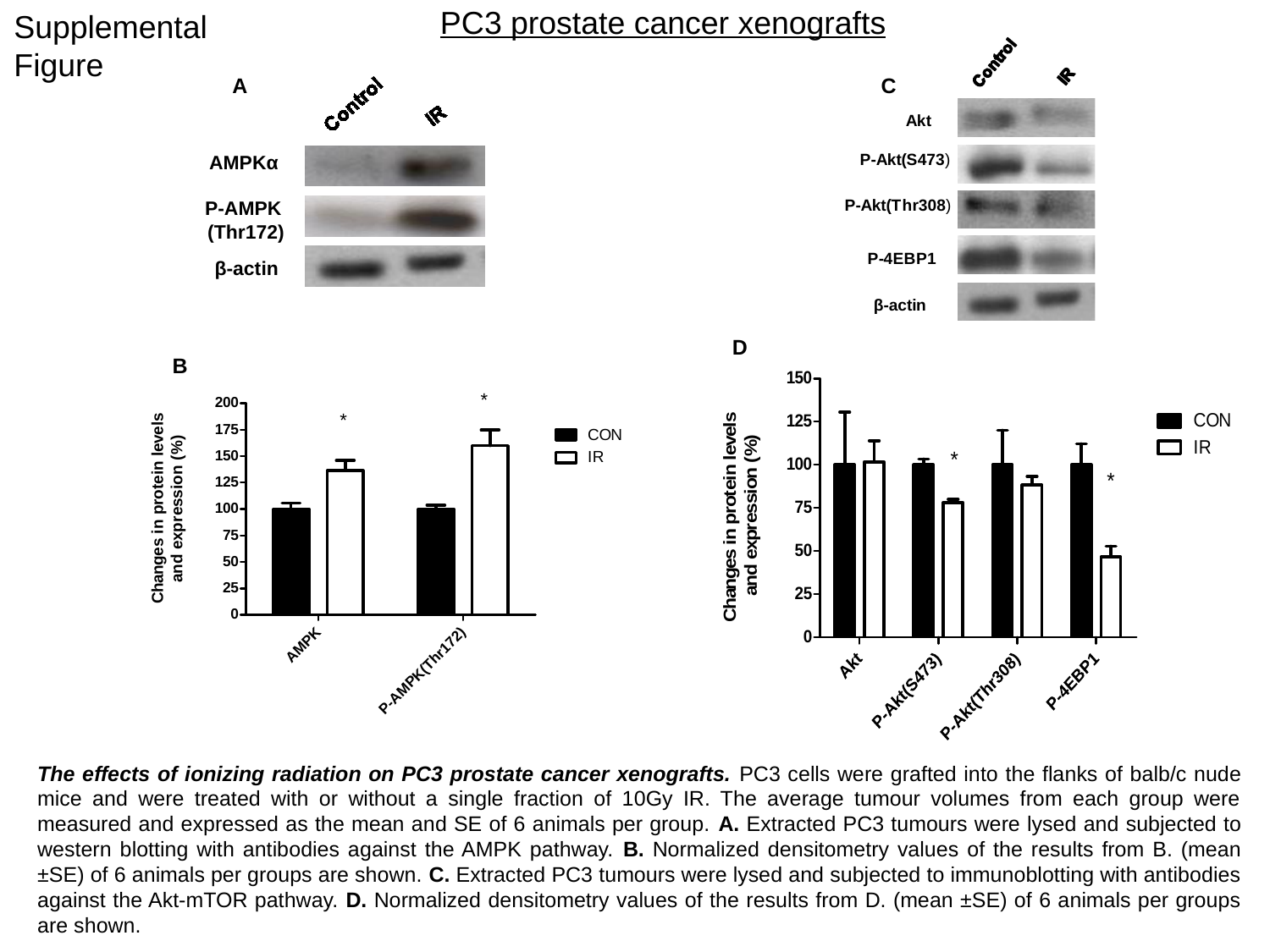

Supplemental
Figure
PC3 prostate cancer xenografts
A
C
AMPKα
P-AMPK
(Thr172)
β-actin
D
B
The effects of ionizing radiation on PC3 prostate cancer xenografts. PC3 cells were grafted into the flanks of balb/c nude mice and were treated with or without a single fraction of 10Gy IR. The average tumour volumes from each group were measured and expressed as the mean and SE of 6 animals per group. A. Extracted PC3 tumours were lysed and subjected to western blotting with antibodies against the AMPK pathway. B. Normalized densitometry values of the results from B. (mean ±SE) of 6 animals per groups are shown. C. Extracted PC3 tumours were lysed and subjected to immunoblotting with antibodies against the Akt-mTOR pathway. D. Normalized densitometry values of the results from D. (mean ±SE) of 6 animals per groups are shown.
